# Household COVID-19 secondary attack rate and associated determinants in Pakistan; A retrospective cohort study

**DOI:** 10.1371/journal.pone.0266277

**Published:** 2022-04-28

**Authors:** Amjad Khan, Muhammad Hassan Mushtaq, Javed Muhammad, Anupam Sule, Ali Akbar, Khunsa Junaid, Ali Akram Khan, Taimoor Akram Khan, Ubaid Khan, Fatmee Waqar, Asghar Khan, Muhammad Akib Warraich, Abdul Jabbar, Abbas Al Mutair, Saad Alhumaid, Maha Al-Mozaini, Kuldeep Dhama, Muhammad Fayaz Khan, Ali A. Rabaan

**Affiliations:** 1 Department of Public Health & Nutrition, The University of Haripur, Haripur¸ Pakistan; 2 Department of Epidemiology and Public Health, The University of Veterinary and Health Sciences Lahore, Lahore, Pakistan; 3 Department of Microbiology, The University of Haripur, Haripur, Pakistan; 4 Department of Informatics and Outcomes, St Joseph Mercy Oakland, Pontiac, MI, United States of America; 5 Department of Microbiology, The University of Baluchistan, Quetta, Pakistan; 6 Department of Community Medicine, King Edward Medical University, Lahore, Pakistan; 7 Department of Clinical Medicine and Surgery, ARID-Agricultural University, Rawalpindi, Pakistan; 8 Department of Marketing, Rennes School of Business, Rennes, France; 9 Department of Medical Lab Technology, The University of Haripur, Haripur, Pakistan; 10 Research Center, Almoosa Specialist Hospital, Al-Ahsa, Saudi Arabia; 11 College of Nursing, Princess Norah Bint Abdulrahman University, Riyadh, Saudi Arabia; 12 School of Nursing, Wollongong University, Wollongong, NSW, Australia; 13 Administration of Pharmaceutical Care, Al-Ahsa Health Cluster, Ministry of Health, Al-Ahsa, Saudi Arabia; 14 Immunocompromised Host Research Unit, Department of Infection and Immunity, King Faisal Specialist Hospital and Research Center, Riyadh, Saudi Arabia; 15 Division of Pathology, ICAR–Indian Veterinary Research Institute, Izatnagar, Bareilly Uttar Pradesh, India; 16 Molecular Diagnostic Laboratory, Johns Hopkins Aramco Healthcare, Dhahran, Saudi Arabia; 17 College of Medicine, Alfaisal University, Riyadh, Saudi Arabia; Konkuk University, REPUBLIC OF KOREA

## Abstract

**Background:**

COVID-19 household transmissibility remains unclear in Pakistan. To understand the dynamics of Severe Acute Respiratory Syndrome Coronavirus disease epidemiology, this study estimated Secondary Attack Rate (SAR) among household and close contacts of index cases in Pakistan using a statistical transmission model.

**Methodology:**

A retrospective cohort study was conducted using an inclusive contact tracing dataset from the provinces of Punjab and Khyber-Pakhtunkhwa to estimate SAR. We considered the probability of an infected person transmitting the infection to close contacts regardless of residential addresses. This means that close contacts were identified irrespective of their relationship with the index case. We assessed demographic determinants of COVID-19 infectivity and transmissibility. For this purpose based on evolving evidence, and as CDC recommends fully vaccinated people get tested 5–7 days after close contact with a person with suspected or confirmed COVID-19. Therefore we followed the same procedure in the close contacts for secondary infection.

**Findings:**

During the study period from 15^th^ May 2020 to 15^th^ Jan 2021, a total of 339 (33.9%) index cases were studied from 1000 cases initially notified. Among close contact groups (n = 739), households were identified with an assumed mean incubation period of 8.2+4.3 days and a maximum incubation period of 15 days. SAR estimated here is among the household contacts. 117 secondary cases from 739 household contacts, with SAR 11.1% (95% CI 9.0–13.6). All together (240) SAR achieved was 32.48% (95% CI; 29.12–37.87) for symptomatic and confirmed cases. The potential risk factors for SAR identified here included; old age group (>45 years of age), male (gender), household members >5, and residency in urban areas and for index cases high age group. Overall local reproductive number (R) based on the observed household contact frequencies for index/primary cases was 0.9 (95% CI 0.47–1.21) in Khyber Pakhtunkhwa and 1.3 (95% CI 0.73–1.56) in Punjab.

**Conclusions:**

SAR estimated here was high especially in the second phase of the COVID-19 pandemic in Pakistan. The results highlight the need to adopt rigorous preventive measures to cut the chain of viral transmission and prevent another wave of COVID-19.

## Introduction

The coronavirus disease 2019 (COVID19) pandemic due to Severe Acute Respiratory Syndrome Coronavirus 2 (SARS-CoV2) has caused a devastating blow to the global economy and population, with the impact of the pandemic still being researched [1,2]. The ongoing pandemic has affected 188 countries around the globe [3]. Most people infected with SARS-CoV2 have clinical conditions that range from asymptomatic to very severe pneumonia. People among high-risk groups are individuals whose age is more than 70 years and have chronic conditions of diabetes and cardiovascular problems [2]. In Pakistan, from 3 January 2020 to CEST, 12 August 2021, there have been 1,080,360 confirmed cases of COVID-19 with 24,085 deaths, reported to WHO. As of 11 August 2021, a total of 39,712,007 vaccine doses have been administered.

A large percentage of these SARS-Cov2 patients isolated and quarantined at home, if the conditions are controlled and kept monitored for until recovery. Hospitalization rate was significantly higher in United States during the covid-19 pandemic [4,5]. In Asia, this rate of hospitalization is low. In Iran, the rate has been estimated at 23 percent [5]. As of 1^st^ February 2021, globally confirmed cases of COVID-19 had reached 103.5 million with over 2.23 million deaths [1]. The virus is transmitted via droplets, and fomites contaminated with aerosol and fecal contamination [3,4]. Previous studies also reported that the transmission efficiency of pre-symptomatic or asymptomatic carriers is about 1/3 of that of symptomatic cases [5,6]. Keeping in mind the transmission dynamics of COVID19, the household contacts may be at serious risk. The transmission of COVID19 in household contacts may result in a resurgence in the COVID19 cases and prolongation of the pandemic. In the initial days, China enforced restrictions on household contacts as the rise in cases was being attributed to household transmission [7]. In an outbreak situation, people are typically recommended to isolate and quarantine infected patients at home in order to control the disease, but this quarantine strategy has less or no effect on transmissibility within the household. Therefore, the quantification of COVID-19 infection risk as the secondary attack rate (SAR) at households is a priority to better understand epidemiologic aspects of this novel disease and the accuracy of measures to prevent viral transmission [6].

People use different mathematical and statistical models to evaluate the transmission pattern at the population level and at the individual level (agent-based models) [8,9]. The transmission pattern and its effect at the household level have not been adequately studied, and there is no detailed report shared to measure the effectiveness of lockdown at the household level [10]. The exposure history from contact tracing at the individual level provides accurate data on the transmissibility of a disease pathogen from human to human. The secondary attack rate is the probability that an infected person will transmit the disease to people at risk, such as household or close contacts. Contact tracing data of an enormous number of cases from China and ten cases from the USA were utilized to estimate the SAR for SARS-CoV-2 [11–13]. However, data pertaining to secondary attack have not yet been published in Pakistan. Understanding important parameters, such as secondary attack rates and direction of transmission, can help align the local public health response with transmission dynamics with the goal of minimizing the morbidity and mortality due to COVID-19. Considering the fact that the Standard Operating Procedures (SOPs) and guidelines issued for COVID19 quarantine have not highlighted the impact of secondary attack, it is important to measure these cases and report them. These estimates give a true picture of confirmed infection proportion among all traced contacts. The main objective of this study is to estimate SAR of SARS-CoV-2 in household close contacts and the individual level exposure and existence of tertiary cases in Pakistan.

## Materials and methods

### Study design

A retrospective cohort study was conducted from 15^th^ May 2020 to 15^th^ January 2021 in the Khyber Pakhtunkhwa and Punjab Provinces of Pakistan. Punjab is the most populated province of Pakistan, while Khyber Pakhtunkhwa is the neighboring province with less population and more diverse climatic conditions. During the study period vaccines were not available against SAR-CoV-2 virus in Pakistan. But current data on (8^th^ Dec 2021) Pakistan has administered at least 127,706,528 doses of COVID vaccines so far. Assuming every person needs 2 doses, that’s enough to have vaccinated about 29.5% of the country’s population. Therefore, the SAR assessed here is purely conducted in non-vaccinated population. Out of 1000 randomly-selected confirmed cases through reverse transcription polymerase chain reaction (RT-PCR). These cases were selected from COVID-19 isolation centers and COVID-19 wards at District level general hospitals, and total of 339 primary index cases were enrolled in the study. These cases were further followed for their close contacts and households through online surveillance, if there is any positive case in their close contacts. Final analysis of 339 patients were conducted; rest were lost due to follow up and most were not having complete record of their close contacts or they were not cooperating through online surveillance used for their follow up.

### Case definition

An individual was confirmed as a case after detection of SARS-CoV-2 using reverse transcription polymerase chain reaction (RT-PCR) [14]. Any individual having laboratory confirmation of SARS-CoV-2 but no clinical signs and symptoms found positive after organization screening, or through contact tracing or community screening, was considered as asymptomatic. Here we analyzed such asymptomatic infections as confirmed cases.

Samples were collected from lower and upper respiratory tract of the suspects in vials or viral transport medium [14,15] and tested for SARS-CoV-2 ribonucleic acid (RNA) by RT-PCR. Depending on the availability of various kits, different kits were used including Abbott Real-time SARS-CoV-2 (Abbott), VIASURE SARS-CoV-2 detection Kit, and Roche Light mix modular SARS-CoV-2 (Roche-TIB MOLBIOL).

### Ethical Approval and Consent

A written informed consent was obtained from all patients before the collection of data. Consent for minors were also obtained from their Parents and Guardians. This study was approved by the departmental review committee of the University of Haripur. Individuals linked by online surveillance tracing contacts were considered a cohort of the close contact group.

### Contact tracing and investigation

The epidemiological investigations were conducted by the Department of Public Health & Nutrition, University of Haripur 24 h after case notification from the designated laboratories. A predesigned questionnaire was used to record demographic, occupation, and diagnostic data for each confirmed SARS-CoV-2 case. Baseline health status and exposure history for 14 days before the appearance of symptoms were also recorded. Such contacts were identified and they were followed for two weeks after first contact with the positive case through RT-PCR with sign and symptoms.

A close contact was defined as an individual who had unprotected close contact, i.e. within 1 meter as per WHO guidelines, with a confirmed case less than two days before onset of symptoms in the patient or sample collection in asymptomatic cases. Close contacts were not limited to household members, but also included contacts in a mosque, church, or other worship places, colleagues working in the same close premises, educational institutes, care centers, regular transport partners, and caregivers. These contacts were tested for COVID-19 by RT-PCR twice on day one and day fourteen. The data were collected as a routine screening program in the community and samples were transferred to the laboratories for diagnostic testing. A case cluster was defined by cases from a similar close contact group. Individuals who resided in or who had visited China or any other foreign country with endemic/epidemic COVID-19 status two weeks before the onset of their symptoms were considered as imported cases. The remaining cases were defined as local cases in the analysis. In the close contact group, amongst the local cases having symptom onset < 1day (for imported cases it was <3 days) from the initial onset day in close contact group were considered as primary case and the rest were considered as secondary cases. Two definitions of household contacts were followed: including family members or individuals who were close relatives, such as brother or sister in law, parents in law or first second or third cousins, regardless of their residential addresses, and those living at the same address irrespective of their relationship within close proximity of the primary case. This was done different from internationally followed standards because the culture in Pakistan is very different. Relatives mentioned here, comes to see the patients and exposes to the patient directly. That’s why people living in the same house were not only considered as close contacts but all the relatives who could come to see the patients were considered close contacts.

### Statistical analysis

Statistical analysis of the data was conducted using SPSS version 26.00. Data were collected on a predesigned questionnaire including demographic and epidemiological questions. Using non-parametric tests including Fisher’s exact test and Mann-Whitney U test to compare the characteristics between the demographic groups. Following [11–13] previously published methods, proportions of the confirmed infections in all the traced contacts and their subgroups were calculated and the SAR was estimated. It was found that the cases were not only secondary necessarily but could be tertiary. The spatial distribution of the cases was analyzed using ArcGIS with a directed graph indicating potential transmission chains.

Reproductive number (Rt) was also calculated using the contact tracing data. The subsequent transmission relationship between the contacts remained unclear, therefore investigation was performed using three different scenarios; (1) The first was that all imported cases were considered primary, and secondary cases were infected by primary cases in the same case cluster. (2) In the second, similar to the first scenario with a difference that the local primary cases might be cases infected by earlier local cases in other case cluster. (3) While in the third scenario, the imported secondary cases were counted as secondary cases instead of primary cases??. The first and third scenarios served as the lower and the upper bounds for Rt. A statistical model called the “chain-binomial model” was utilized to estimate the SAR and the local reproductive number (Rt). The possible distribution of the infection period and the incubation period was derived from previously published literature [18–20]. Following available literature [8,16], we reported estimates allied with the mean incubation period of 5–13 days minimum to maximum as the primary results.

The model we used in this study estimated probabilities including *p*1 and *p*2, for the viral transmission from infectious household contacts and from the infectious non-household contacts (colleagues, passengers, prayers contact in a mosque, and during sports at the local level) respectively to other susceptible individuals per daily contact. We also assumed that COVID-19 case infectivity differs between the incubation period and after symptom period onset (illness period). For this purpose, we used (D_min_ and D_max_) to calculate the whole infectious period with symptom onset days considered as day 0, and modeled effective daily transmission likelihood as

pk(l)=pkondayDmin≤l<0(theincubationperiod)and

pk*(l)/1–pk(l)=pk/1–pk×ORonday0≤l<Dmax(theillnessperiod),

Here the OR (Odds Ratio) measures the infectivity of the illness period versus the incubation period. The SAR was calculated as follow:

1–_l=DDmaxmin[1−pk*(l)_*(l)],k=1,2,

Here, φ*(l) is the prespecified relative infectivity level on the first day of the infectious period based on the previous studies [19] peaking near symptom onset time.

Herein, as model based estimates SAR was derived. The local Rt was defined as (for household and non-household):

$$\sum^{2}k=\sum 1‐l=D\max\;D\;mim\;\{nk(l)pk(l)‐(l)‐m=D\;2\min\;l–1*\;*[1‐p*k(l)‐*(l)]\}$$

Where n1 (l) and n2 (l) are mean numbers of the household and the non-household contacts per primary cases on day l [17].

We also determined the effect of gender, age, epidemic phase, and household size. It was performed for infectivity and susceptibility by regressing the transmission of the probabilities on these factors associated with susceptibility of the person or the infectious individual in each of the potential transmission-exposure pair. Goodness of fit of the model was also assessed. In univariate analysis, Poisson regression was used at a confidence interval of 95%. Dependent variable was SAR and independent factors studied as potential risk factors included, gender, age, number of the household members, and their relevant residence either urban or rural and semi-urban. Directed Acyclic Graphs (DAG) were used to assess the relationships among the secondary cases versus independent variables with the number of household contacts considered as exposure and the secondary cases as their outcome following previous literature [8,16].

## Results

Randomly 339 index cases were enrolled in the study (33.9% of 1000 cases initially tested and followed) from the list of COVID-19 Positive patient’s directory using it as a sampling frame for our study. The Response rate (61%) from participants was considered satisfactory. One hundred and seventeen laboratory confirmed COVID-19 secondary cases were found in 739 household contacts that become 15.83% SAR among the enrolled participants. Other than these confirmed cases, 213 (28.8%) acute symptomatic infections were also recorded in the household contacts. These could also be secondary COVID-19 cases based on symptoms. A SAR of 32.4% is obtained by considering symptomatic and confirmed cases together. The incubation period was estimated by finding the difference of the onset day between the secondary cases and index cases. The median calculated here was 7 days (range 3–15) with a mean of 8.2+4.3 days.

Details of the household contacts and index cases are shown in Table 1. Here household contacts (35.6 + 22.1) were younger as compared to index cases (49.7 + 18.4). The male-female ratio was almost similar in both household contacts cases and index cases. Almost half of the study population were living in a joint family system having an average of >5 members living together. More than half of the secondary and index cases enrolled, we recorded that hospitalization was lower in secondary cases than index cases while pneumonia and mortality were higher in index cases than secondary cases.

**Table 1:** Description of Covid-19 households and their index cases in Khyber Pakhtunkhwa and Punjab Province of Pakistan, May 17th, 2020—January 15th, 2021.

Variable	Household Contacts N = 739 (%)	Index cases N = 339 (%)	P-values
**Age (Mean + SD)**	35.6 + 22.1*	49.7 + 18.4	0.031
**Gender**			
Male	465 (62.92)	211 (62.24)	<0.001
Female	274 (37.08)	128 (37.76)	
**No of households members**			
1 to 3	160 (21.65)	81 (23.90)	<0.001
4 to 6	218 (29.49)	107 (31.57)	
> 7	361 (48.85)	151 (44.53)	
**Province (Case Origin)**			
Khyber Pakhtunkhwa	421 (56.97)	187 (55.16)	0.002
Punjab	318 (43.03)	152 (44.84)	
**Residence (at first sign)**			
Urban	512 (69.29)	262 (77.29)	<0.001
Rural	227 (30.71)	77 (22.71)	
**Secondary Cases**			
Laboratory Confirmed	117 (15.83)	-	
symptoms based	213 (28.83)**	-	
All together	240 (32.48)	-	
**Cases Outcome**			
Hospitalized	72 (61.53)^	153 (45.13)	0.001
Pneumonia	39 (33.33)^	137 (40.41)	
Mortality	6 (5.12)^	22 (6.48)	

*Age missing for 11 household contacts.

** Symptoms data missing for 9 household contacts.

^Percent of 117 confirmed secondary cases.

Univariate analysis of the data was conducted to identify the potential risk factors of secondary COVID-19 cases found (Table 2). Several potential risk factors were found including; COVID-19 positive patient’s age, male gender, household members seven or more than seven, and residency at urban area. From index cases cough, pneumonia and hospitalization were the important risks. The risk was found low in relation to several variables: gender male, residency in rural area and young age group. The incidence rate of acute COVID-19 infection symptoms was higher in the old age group >45 years with a significant difference.

**Table 2:** Confirmed secondary COVID-19 cases risk factors analysis through Univariate Poisson regression in the Province of Khyber Pakhtunkhwa and Punjab, Pakistan, Feb 26th-April 8th, 2020.

Variable	Secondary confirmed cases n = 117	No cases n = 622	RR	95% CI	P value
Age + SD*	57.2 + 8.3	43.7 + 13.4	1.17	0.97–1.61	<0.001
Ages groups (years)*					
0–25	0	341	-	-	-
26–40	18	187	15.3	2.13–25.1	0.003
41–60	67	42	31.4	5.22–53.3	<0.001
>61	32	52	27.1	4.31–44.7	0.001
**Gender**					
Male	77	388	1.16	0.72–1.65	0.021
Female	40	234	-	-	-
**No of household members**					
1 to 3	11	149	-	-	-
4 to 6	40	178	1.67	0.91–2.51	0.031
> 7	66	295	5.12	2.89–7.21	<0.001
**Residence**					
Urban	79	433	1.08	0.41–1.89	0.031
Rural	38	189	-	-	-
**Index case**					
Age + SD	-	-	1.03	0.62–1.53	0.044
**Gender**					
Male	81	384	1.02	0.03–1.92	0.027
Female	36	238	-	-	-
**Cough**					
Yes	92	412	2.11	1.18–4.37	0.033
No	25	210	-	-	-
**Pneumonia**					
Yes	34	248	1.18	0.88–2.01	0.004
No	83	374	-	-	-
**Hospitalization**					
Yes	41	279	1.83	0.98–2.82	<0.001
No	76	343	-	-	-

**Fig 1:**
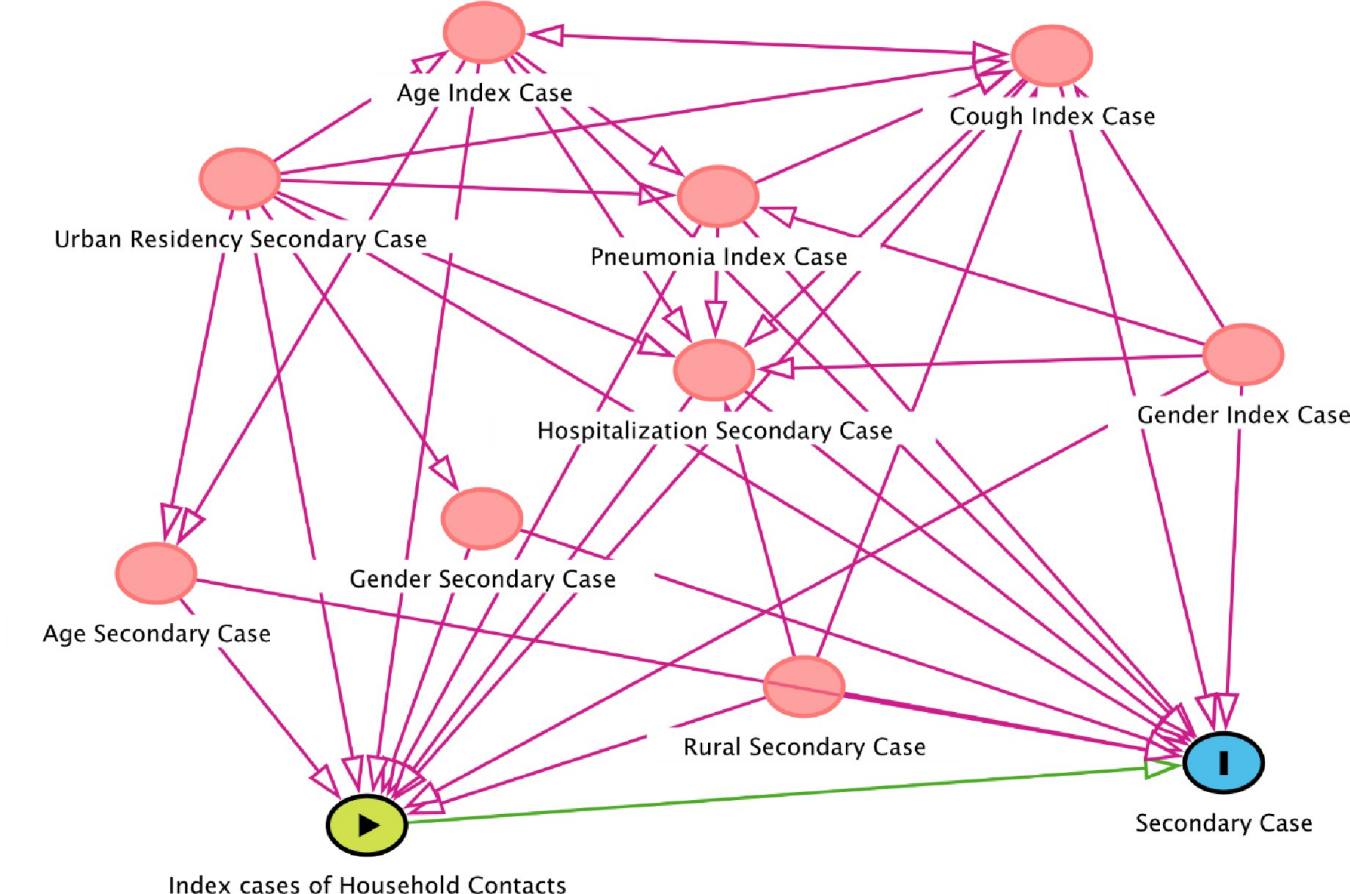
Directed Acyclic Graph (DAG) of Pakistani household index case (exposure) contacts effect on the COVID-19 secondary cases (outcome). Ancestors of outcome (red color) and exposure.

The potential risk factors were also studied using Directed Acyclic Graphs (DAGs) approach (Fig 1). Variables were adjusted through different covariates with the inverse probability weighting (Table 3) adopting the previously used method [18]. Old age groups of secondary cases, household members more than 5, being resident in an urban area, gender being male, and in index cases, higher age had elevated risk. Cough, pneumonia, hospitalization, and rural area of index cases lost association with the disease.

**Table 3:** Relative risk and adjusted SARs of COVID-19 secondary case using an inverse probability weighting in Pakistani household contacts.

Variable	Adjusted SAR (%)	Adjusted Relative Risk (RR)	95% Confidence Interval	P value
**Ages groups (year)** ^**I**^				
0–25	0.51	1.00	-	-
26–40	3.73	8.44	2.14–21.07	0.015
41–60	10.3	28.2	5.11–32.47	0.001
>61	21.8	42.5	13.1–62.5	<0.001
**Gender** ^**II**^				
Male	7.21	4.88	1.71–6.23	0.003
Female	3.70	1.00	-	-
**No of household members** ^**III**^				
1 to 3	2.12	1.00	-	-
4 to 6	9.32	3.87	2.23–4.91	0.008
> 7	13.11	5.43	3.41–6.11	0.001
**Residence** ^**IV**^				
Urban	3.67	2.15	1.70–3.09	0.006
Rural	1.32	1.00	-	-
**Index case**				
**Cough** ^**V**^				
Yes	5.21	2.87	1.11–3.98	0.002
No	1.07	1.00	-	-
**Pneumonia** ^**V**^				
Yes	7.21	3.77	2.20–4.57	0.024
No	1.71	1.00	-	-
**Hospitalization** ^**V**^				
Yes	4.12	1.78	1.12–2.79	0.003
No	3.11	1.00	-	-

^I^ Adjusted for the index case age.

^II^ Adjusted for household contacts gender.

^III^ Adjusted for household contacts members.

^IV^ Adjusted for household residence.

^V^ Adjusted for index case (hospitalization, pneumonia, and cough).

Following Raad et al., 2020, Inverse probability weighting technique is used to assess the to estimate the probability of the exposure observed for a particular person, and using the predicted probability as a weight in subsequent analyses.

## Discussion

The index cases studied here, followed by their secondary cases, suggest that the household contacts of COVID-19 index cases are at higher risk of getting the disease, seeing that the included cases are only laboratory confirmed. Thus, detection of secondary infections should be performed aggressively in household contacts. Coronavirus attack rate is estimated to be 2.3 per 100 000 Pakistani population. Nearly 49% of cases are registered from Punjab at the time period of this study. Current status (8^th^ December 2021) is that total of 1.2 million confirmed cases have been identified, with 0.02 million deaths, and 1.2 million infected individuals have been recovered. Total tests performed till now are 22.3 million. Vaccination doses given till now are 82.22 million as first dose and 53.7 million individuals are fully vaccinated till now. Including the acute COVID-19 symptoms, an increase in the attack rate was observed in the Pakistani population. The attack rate was significantly higher in Punjab province as compared to Khyber-Pakhtunkhwa. An urban place of residence increases the risk by twofold compared to the rural population. The male population is at higher risk than females. This might be because most of the female population had less exposure to the outside environment and people in terms of contact. They are predominantly housewives and a small number of females work outside their residence. The SAR estimated in our study is almost similar to recent studies reporting 7.5–30% [14–20]. In most of these studies, PCR was used for diagnosis. The SAR in China (30%), and France (73%) was more elevated, but in the United States it was lower (10.5%). The transmission rate of household clusters (20.5%), in COVID-19 close contacts cases (11.7%) and transmission from family members (31%) is similar to prior published articles [15,18]. In our study, the index cases appeared highly contagious in the first 7 days (range 5–9) after the onset of symptoms [21]. The difference in the transmission rate and SAR may be due to the genetic variability of the virus and also the immune response of populations from different nations. It was also observed that the SARs in the household contacts has risen from 4% to 37% in comparison to the 2009 pandemic [22].

Risk factors analysis was also conducted; where the potential risk factors were older age, presence of household members >5, residence in urban areas, male gender, and older age of index cases. Urban areas are mostly affected due to high population density and crowding in different areas. A higher number of family members is at higher risk due to the reason of more contact and exposure to index cases. The male gender seems to be at higher risk because most of the working population in Pakistan are males. Due to higher exposure males were infected more than females.

In Pakistan, the adult population was found to be the main agent of transmission of the disease. If the pandemic persists, surveillance in children needs to be started to understand the dynamics of COVID-19 transmission. The symptoms for acute infection in our study in the different age groups were found similar in older age >35 years. Low risk of secondary cases recorded by spouses of the index cases in Pakistan is reportable which might be due to the reason that the female gender in our study seems to be at low risk of getting an infection as compared to males. In previous studies, a high risk and transmission rate has been reported for the spouses [15,22]. Pneumonia, cough, and hospitalization of the index cases are the main markers of COVID-19 severity in the univariate analysis associated with the secondary cases. But after adjusting for various variables, associations are lost. Several factors contributed to this, including the government policy regarding the control and prevention of COVID-19 from time to time. Smart lockdown (a special type of lockdown in which movements are restricted and banned in high incidence areas of COVID-19) practice in Pakistan seems to have played an important role in the dynamics of disease transmission in both phases (in April and December mostly) of the pandemic. The general population may have been better compliant to the guidelines issued by the government. Several other factors might have contributed to the lower SAR in Pakistan in comparison to India and Iran [5]. These factors may include the difference in the virulence of the virus and the immune response of the population. Secondary COVID-19 cases were obtained through laboratory confirmation, history, hospitalization, and conducting interviews of household contacts and patients. Risk factors were estimated by adjusting for various variables which permit high precision.

The findings in this study report high SAR in Pakistani household contacts though less than other countries reported previously [21–25]. The results highlight the need of preventive measures at homes considering the high rate of transmissibility of COVID-19 [26] and potential transmission through the fecal-oral route [27,28]. Screening tests are important to be conducted especially for household contacts being a high-risk population [29].

The study’s limitations included; screening tests of household members were not conducted at a large scale, which was important to find more secondary cases, as asymptomatic and mild clinical cases exist in COVID-19 contacts [23]. Other factors were also needed to be studied that may play a role in the transmission of infection such as socioeconomic status and environmental condition of the home. Here we can add that susceptible person should consult lab/hospital to rule out the COVID-19 infection to avoid SAR.

## Conclusions

The estimated SAR among COVID-19 household contacts was found elevated with an increase in age, residency at urban areas, householders with > 5 members. To prevent the spread and reoccurrence of infection preventive measures needed to be taken for a prolonged period. Serological surveys are very important to be conducted to understand the level of natural herd immunity of the population achieved. It will also identify the timeline and importance of vaccination against COVID-19.
